# A family of Fe-N-C oxygen reduction electrocatalysts for microbial fuel cell (MFC) application: Relationships between surface chemistry and performances

**DOI:** 10.1016/j.apcatb.2016.12.013

**Published:** 2017-05-15

**Authors:** Carlo Santoro, Alexey Serov, Rohan Gokhale, Santiago Rojas-Carbonell, Lydia Stariha, Jonathan Gordon, Kateryna Artyushkova, Plamen Atanassov

**Affiliations:** Department of Chemical & Biological Engineering, Center for Micro-Engineered Materials (CMEM), University of New Mexico, Albuquerque, NM 87131, USA

**Keywords:** PGM-free, Power generation, Surface chemistry, Microbial fuel cell, ORR

## Abstract

Different iron-based cathode catalysts have been studied for oxygen reduction reaction (ORR) in neutral media and then applied into microbial fuel cells (MFC). The catalysts have been synthesized using sacrificial support method (SSM) using eight different organic precursors named Niclosamide, Ricobendazole, Guanosine, Succinylsulfathiazole, Sulfacetamide, Quinine, Sulfadiazine and Pyrazinamide. Linear Sweep Voltammetry (LSV) curves were obtained for the catalysts using a O_2_ saturated in 0.1 M potassium phosphate buffer and 0.1 M KCl solution and a Rotating Ring Disk Electrode (RRDE) setup in order to study the ORR characteristics. Additionally, we analyze the peroxide yield obtained for each catalyst which helps us determine the reaction kinetics. Those catalysts have been mixed with activated carbon (AC), carbon black (CB) and PTFE and pressed on a metallic mesh forming a pellet-like gas diffusion electrode (GDE). Results showed that Fe-Ricobendazole, Fe-Niclosamide and Fe-Pyrazinamide had the highest cathode polarization curves and highest power densities output that was above 200 μWcm^−2^. Fe-Ricobendazole, Fe-Niclosamide, Fe-Pyrazinamide, Fe-Guanosine Fe-Succinylsulfathiazole and Fe-Sulfacetamide outperformed compared to Pt cathode. Fe-Sulfadiazene and Fe-Quinine performed better than AC used as control but less than Pt. Correlation of surface composition with performance showed that power density achieved is directly related to the total amount of nitrogen, and in particularly, N coordinated to metal and pyridinic and pyrrolic types while larger amounts of graphitic nitrogen result in worse performance.

## Highlights

• Novel precursors were used to fabricate iron based catalysts.
• RRDE current was well correlated with cathode polarization current and power generated.
• Several Fe-based catalysts outperformed Pt.
• Fe-Ricobendazole, Fe-Niclosamide and Fe-Pyrazinamide had power density above 200 μWcm^−2^.
• Power density is directly related with N coordinated to metal and pyridinic and pyrrolic types.

## Graphical abstract

Several organic precursors have been used to fabricate Fe-based catalysts using sacrificial support method. Those catalysts were then included in air breathing cathodes for microbial fuel cells working at neutral environment. Electrochemical performances and surface chemistry were measured and related.

**Figure:**
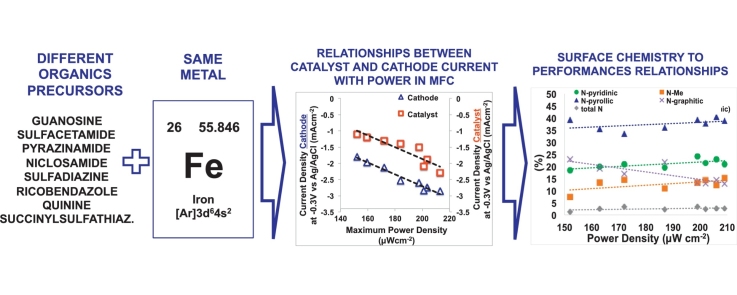

## 1 Introduction

Microbial Fuel Cell (MFC) is a promising technology for combining cleaning of wastewater and generating useful electricity [1]. Several studies have been reported focusing on increasing electricity output and efficiency of organics removal [2].

Particular effort has been invested into the research and developments of novel materials for anode and cathode [1]. Different conductive materials have been studied as anode electrodes ranging from various metals (e.g. copper, silver, cobalt and titanium) [3] to stainless steel [4], [5] with relatively promising results. Three-dimensional carbonaceous materials still remain the most utilized as anode due to its beneficial 3D structure, relatively high conductivity, and low cost [6], [7]. It should be noticed that performance at the cathode is still limiting power/current output in MFC.

Low cathode catalytic activity in oxygen reduction reaction (ORR) [8] and relatively high cost of the catalytic materials [8] are restricting MFC scalability. It was shown that inorganic ORR catalysts suffer from high overpotentials at neutral pH due to the intrinsic nature of the mechanism of the oxygen reduction reaction [8]. On the other hand, enzymatic catalysts and, particularly, bilirubin oxidase and laccase showed the lowest overpotentials at pH close to 7 [9], [10], [11]. Unfortunately, the low density of active sites [10], [11], the low durability in “clean” (model ORR reaction in buffered electrolyte), and “dirty” (real MFC operation in presence of contaminants) conditions [12], and the relatively high cost compared to the power generated do not allow their implementation at the large scale as well as for long operation times.

Carbon-based catalysts have also been extensively adopted for MFCs cathode [7], [13]. It has been shown that activated carbon, carbon nanofibers and carbon nanotubes have high surface area, electrical conductivity, mechanical strength and durability that are important properties to ensure high activity in oxygen reduction in neutral media [14], [15], [16], [17], [18], [19], [20]. It is well known that platinum is one of the most active electrocatalysts for electroreduction of oxygen in acidic media [21], however the main drawbacks related to platinum utilization are: i) high cost and relatively low abundance; ii) low activity at neutral pH; iii) poisoning in harsh environments and, in particular, in presence of sulfur-containing species.

Taking into account the disadvantages of platinum, several research groups shifted their paradigm towards the utilization of low cost and widely available base metals like iron, cobalt, and manganese that can be atomically dispersed within the structure of the catalyst while not affecting the cost of the final material. These materials also show high electrocatalytic activity to the electroreduction of oxygen [22], [23], [24]. In particular, iron-based catalysts have been recently utilized as cathode catalyst in MFC by few groups [25], [26], [27], [28], [29], [30], [31], [32]. Recently cobalt-based catalysts have also been used as ORR catalysts to enhance MFC performances [25], [33], [34], [35], [36], [37], [38]. At last, manganese dioxide based catalysts showed impressive electrocatalytic activity at neutral pH comparable to or higher than platinum-based catalysts [39], [40], [41], [42]. It should be noticed that iron-, cobalt-, and manganese-based catalysts have been previously tested in acid [43] or alkaline [44] media where the high presence of H^+^ or OH^−^ resulted in higher oxygen reduction kinetics. The superiority of such catalysts compared to platinum-based materials, especially in alkaline media, has been observed [45], [46].

In previous studies, we showed that Fe-Aminoantipyrine [47], Fe-Niclosamide, and Fe-Ricobenzadole [48] prepared with sacrificial support method had excellent performance in neutral media and in MFC. Particularly, Fe-Aminoantipyrine has been tested in: i) double chamber MFC [49], ii) single chamber MFC [47] and iii) ceramic based MFC [50]. Furthermore, Fe-Niclosamide and Fe-Ricobendazole were recently tested in single chamber MFC for 32 days [48]. Power densities slightly around 200 μWcm^−2^ were recorded and presented and are among the highest reported in the open literature using those operating conditions [48]. Fe-Ricobendazole and Fe-Niclosamide showed much higher stability compared to Pt in long terms experiments [48].

Herein, we report the performances and the surface structural properties of eight low-cost iron-based catalysts synthesized using sacrificial support method [51], [52]. The ORR of those novel catalysts were studied using rotating ring disk electrode (RRDE) to investigate the catalysts kinetic, the electron transfer mechanism and the production of hydrogen peroxide (H_2_O_2_) during the polarization. The RRDE performances of all those Fe-catalysts have not been presented yet. These highly active catalysts were then incorporated into a gas diffusion electrode (GDE), air-breathing type, by making a hybrid of activated carbon, carbon black and PTFE and pressed on a metallic support. Among those Fe-based catalysts, six have been synthesized from novel low-cost organic precursors (Guanosine, Succinylsulfathiazole, Sulfacetamide, Sulfadiazine, Quinine, and Pyrazinamide) and were not previously reported, while systematic study of catalysts based on Niclosamide and Ricobendazole precursors incorporated in air-breathing cathodes was previously presented but RRDE and surface to properties analysis were not studied [48]. It was decided to include Fe-Ricobendazole and Fe-Niclosamide as catalysts also into the current work to enhance the number of samples studied and make stronger statistical correlations. In this experimentation, power densities above 200 μWcm^−2^ were achieved by Fe-Ricobendazole, Fe-Niclosamide and Fe-Pyrazinamide. Direct relationships between RRDE current ouput, current output from the catalysts incorporated in air-breathing cathode and MFC power/current output are here presented. Moreover, the relationship between power generation in MFCs and the surface chemistry of the catalyst are described in this report. Positive effect of N-pyridinic, N-pyrrolic and N coordinated to metal (N_x_-Me) on power generated is illustrated. It is shown that several low-cost iron-based electrocatalysts have very high performance and can be utilized successfully for large-scale MFC configurations.

## 2 Materials and methods

### 2.1 Fe-N-C catalysts synthesis by SSM

The Sacrificial Support Method was modified in order to increase the pore size of materials and decrease the overall surface area to the ∼600 m^2^ g^−1^. The mixture of two types of sacrificial support was used: i) medium surface area (SA) LM150 (SA = 150 m^2^ g^−1^) and ii) low SA OX50 (SA = 45 m^2^ g^−1^). After making a colloidal solution of mentioned above silicas organic precursors dispersed in water was added, followed by addition of FeNO_3_*9H_2_O (Sigma Aldrich). The stirring hot plate was heated to 45 °C and water was allowed to evaporate resulting in a viscous paste. The rest of water was evaporated at T = 85 °C, solid materials were ground in a mortar and used for high temperature treatment. The HT temperature was selected as 975 °C with a fast ramp rates of 10 °C min^−1^. Ultra-high purity (UHP) nitrogen with flow rate 100 ml min^−1^ was used in all experiments. After pyrolysis mixture of silica materials was removed by etching in 25 wt% solution of HF. Black powders were carefully washed with DI water and dried at T = 80 °C. The low cost organic precursors used were: Guanosine, Succinylsulfathiazole, Sulfacetamide, Sulfadiazine, Pyrazinamide, Quinine, Niclosamide and Ricobendazole.

### 2.2 Materials surface analysis

Scanning Electron Microscope (SEM) was used to show the morphology of the novel synthesized catalysts. The images provided useful information about the bulk and the single particle morphology. Surface chemistry has been investigated using X-ray photoelectron spectroscopy (XPS). Three different areas of each catalyst were investigated. Measurements were taken using a Kratos Axis Ultra DLD XPS with a monochromatic Al Ka source that operated at 225 W. For this work, C1s, O1s and N1s were of interest and they have been acquired at pass energies of 20 eV. Charge compensation was not utilized since samples were conductive. CASAXPS software was used for analyzing the data and quantifying the contribution of C1s, N1s and O1s. The data were quantified using specific sensitivity factors provided by the manufacturer. The curves were fit using a 70% Gaussian/30% Lorentzian [GL(30)] line shape.

### 2.3 Study of the kinetics of the Fe-catalysts using RRDE

Linear sweep voltammetry data was obtained for all the catalysts for oxygen reduction currents at the disk and peroxide oxidation currents at the ring using a rotating ring disk electrode (RRDE) setup. The working electrode was a glassy carbon disk with an outer concentric platinum ring. Graphite rod was used as the counter-electrode and Ag/AgCl (3 M KCl) as the reference electrode. The electrolyte was a O_2_ saturated buffer solution (0.1 M K-PB and 0.1 M KCl) with a pH of 7.5. The catalyst inks were prepared by adding 2.5 mg of the catalyst to 150 μL of 0.5 wt% Nafion solution and 850 μL of water: isopropanol mixture (4:1 by volume). The inks were sonicated before drop-casting on to the working electrode-disk (a total loading of 100 μg cm^−2^). The rotation speed of the working electrode for the measurements was 1600 rpm. The potential of the ring was maintained at 0.7 V vs Ag/AgCl.

### 2.4 Cathode preparation

Air breathing gas diffusion electrodes (GDEs) were used during the electrochemical characterization. Particularly 70 wt% activated carbon (AC, SX Ultra, Sigma Aldrich), 20 wt% PTFE (60 wt% solution, Sigma Aldrich) and 10 wt% carbon black (CB, Alfa Aesar) were firstly mixed into a dispersing blender for 2 min and weighted before being inserted into a metallic pellet die. Carbon black was used to enhance the electrode conductivity [53]. Each iron-based (Fe-N-C) catalyst was then mixed separately with the black powder (AC/CB/PTFE) previously blended. The obtained mixture was then pressed using a professional press (Carver, USA) at 3 mT for 5 min as previously reported [54]. AC/CB/PTFE had a loading of 40 ± 1 mg cm^−2^ and the catalyst had a loading of 2 ± 0.1 mg cm^−2^. No heating treatment has been applied. Platinum-based cathode and AC cathode were used as a control for the performance comparison. In particular, in the case of Pt catalyst, the mentioned above mixture of AC, CB and PTFE (70/10/20 wt%) was separately prepared and 50 wt% Pt/C (Alfa Aesar, USA) was then blended with the mixture followed by pressing at the same conditions: 3 mT (metric tons) for 5 min. To be consistent with the Fe-based cathodes and to have a fair comparison, AC/CB/PTFE had a loading of 40 ± 1 mg cm^−2^ and the Pt loading was 2 ± 0.1 mg cm^−2^. AC cathode was fabricated without the addition of any catalyst and AC + CB + PTFE (70/10/20 wt%) loading was 40 ± 1 mg cm^−2^.

### 2.5 Cell design and testing

Cathodes were screwed to a lateral hole of a single bottle MFC with a volume of 125 mL and with the geometric cathode exposed area to the liquid solution of 2.8 cm^−2^. Screening cathode polarization curves in duplicate were done in potassium phosphate buffer solution (K-PB) and 0.1 M KCl at pH = 7.5 after exposing the cathode to the solution overnight (at least 16 h) and observing a stable OCP. No polymer exchange membrane was used in membraneless configuration. After initial polarization curve in “clean” conditions, the cathode was then inserted in a single chamber MFC with pre-colonized anode that has been working for over 6 months continuously [54]. The MFC was then left in Open Circuit Voltage (OCV) for at least 6 h. The operating solution was based on K-PB (0.1 M) and KCl (0.1 M) + activated sludge (50% in volume each) and 3 gL^−1^ sodium acetate. The activated sludge was collected from the Albuquerque Southeast Water Reclamation Facility (Albuquerque, NM, USA). The anode was a carbon brush (Millirose, USA) with a diameter of 3 cm and length of 3 cm. Anode projected area was 9 cm^2^ [54]. The experiments were performed in Albuquerque (NM, USA), which is located at high altitude (≈1600 m above sea level) compared to sea level, and this effect of the atmospheric partial pressure as well as the oxygen partial pressure negatively affect the cathode performances.

### 2.6 Electrochemical measurements of the catalyst incorporated into air breathing cathodes

Cathode linear sweep voltammetry (LSV) was run using a three-electrode configuration with a cathode as working electrode, Ag/AgCl 3 M KCl (+0.21 V vs SHE) as reference electrode and a Pt mesh as counter electrode. The cathodes have been tested in the range between OCP and −0.35 V vs Ag/AgCl at a low scan rate of 0.2 mV s^−1^. Before starting the experiments, the cathodes have been left overnight in direct contact with the solution till the OCP was stable. That value has been considered as cathode OCP during further analysis. MFC polarization curves were recorded using a potentiostat (Biologic, France) connecting the anode as working electrode, the cathode as counter and a Ag/AgCl (3 M KCl) as reference electrode. This set up allowed the separate registration of the anode and cathode profiles along the overall polarization curve. In order to obtain the reproducible results, all the measurements were performed at least in duplicate. The power generated was calculated by multiplying voltage and current measured. Power density and current density were reported by dividing the value obtained for the cathode geometric area (2.8 cm^−2^).

### 2.7 Surface to performance analysis

Surface chemistry and performances were correlated with the utilization of principal components analysis (PCA) which is a powerful statistical tool utilized for processing large data sets with the purpose of identifying direct and reverse relationships between variables. The dataset analyzed in this work consists in the electrochemical performances output (power density, current density during LSV, current density during RRDE and %H_2_O_2_) and in the surface chemistry (amount of N, N-pyridinic, N-Me, N-pyrrolic, and N-graphitic) determined by XPS data. These experimental data were included as variables into the data matrix and surface-to- performance relationship were derived. PCA clearly shows the correlations and anti-correlations among the different samples and variables through creation of uncorrelated mathematical components from linear combination of original variables that are called principal components. PCA has been previously successfully adopted by our group [55].

## 3 Results and discussion

### 3.1 Morphology and surface chemistry

**Fig. 1:**
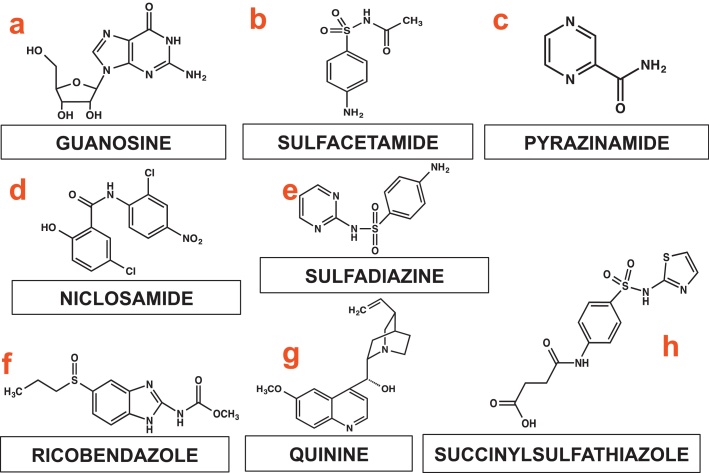
Structural formula of organic precursors: Guanosine (a), Sulfadiazine (b), Pyrazinamide (c), Niclosamide (d), Sulfacetamide (e), Ricobendazole (f), Quinine (g) and Succinylsulfathiazole (h).

The structural formula of the eight organic precursors used as precursors for the catalysts synthesis is shown in Fig. 1. It must be noted that nitrogen is a critical element in the structure of these organic precursors. It was shown previously in acidic and alkaline media that C—N bonds are necessary for making an effective and efficient catalyst for ORR [51], [52], [56].

**Fig. 2:**
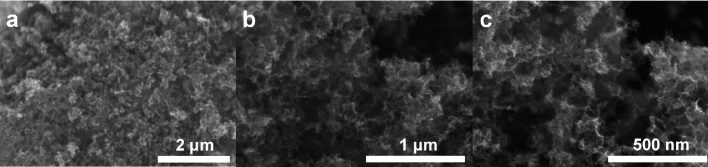
SEM images at different resolutions of Fe-Ricobendazole.

Morphology of the catalyst was obtained through SEM images at different resolutions (Fig. 2). Fe-Ricobendazole was showed as example of the catalysts investigated. The images acquired were similar to previously reported catalysts obtained though sacrificial method support method (SSM) [51], [52], [56]. It must be noted that two scales of pores size were found: the first one between 50 and 100 nm, that is probably due to the sacrificial support (silica) removal and the second one that was much smaller and ranged between 5 and 10 nm that is probably due to organic precursor molecules decomposition and pore-forming action.

Surface analysis of all catalysts was performed by X-ray Photoelectron Spectroscopy (XPS). The overall amount of nitrogen is quite similar between 2 and 3 atomic percent. The distribution of different types of nitrogen species was typical for M-N-C obtained by SSM [56]. Pyridinic nitrogen and nitrogen coordinated to metal, which have been shown to be important species for ORR in acidic media [56] are detected in significant amounts as shown in Table 1. The largest spread in values among the catalysts is observed for pyrrolic and graphitic nitrogen.

### 3.2 RRDE results

**Fig. 3:**
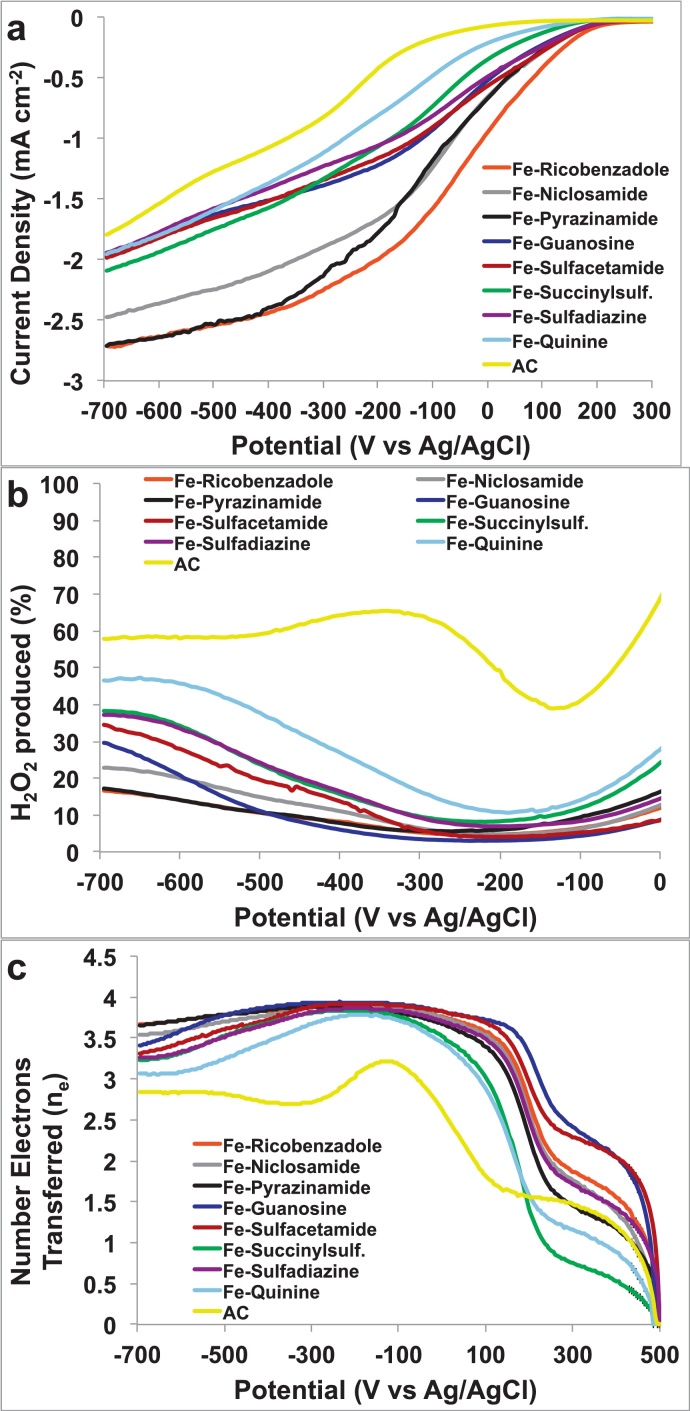
(a) LSV of Fe based catalysts in comparison with AC in O_2_ saturated electrolyte (0.1 M K-PB and 0.1 M KCl) at a rotation rate of 1600 rpm. (b) % H_2_O_2_ produced by the catalysts at different potentials (c) number of electrons transferred in the ORR kinetics of the Fe-catalysts.

Fig. 3a shows the LSV curves obtained for oxygen reduction reaction for all the catalysts. The parameters that define catalytic performance are usually considered as the onset potential of catalysis, half wave potential of the LSV and the saturation current density. It is clear that all the Fe based catalysts show improved performance as compared activated carbon (AC) in all parameters of performance (Fig. 3a). Fe-Ricobendazole exhibits the best catalytic performance in comparison to other catalysts in terms of the most positive potential of −5.7 mV vs Ag/AgCl at current density of 1 mA cm^−2^, highest current density of ∼2.6 mA cm^−2^ and a reasonable onset potential for neutral media electrolyte of ∼234.6 mV vs Ag/AgCl. Fe-Pyrazinamide and Fe-Niclosamide exhibit the next best catalytic properties with potentials of −62.3 and −59.9 mV vs Ag/AgCl at current density of 1 mA cm^−2^, respectively. The worst performing were Fe-Sulfadiazine and Fe-Quinine respectively despite higher performances compared to AC.

Fig. 3b demonstrates the %H_2_O_2_ obtained in all catalysts, derived from calculations from the ring current in RRDE studies (Eq. (1)).

(1) $$\%H_{2}O_{2}=\frac{200\times \frac{i_{ring}}{N}}{I_{disk}+\frac{I_{ring}}{N}}$$

All the Fe-based catalysts show much less peroxide production as compared to AC which exhibits a huge 64.2% peroxide generation at −300 mV vs Ag/AgCl. This is proof of the fact that an inefficient 2-electron oxygen reduction process takes place in AC resulting in large peroxide production. In constrast and consistent with the RDE data, the least H_2_O_2_ production is observed in Fe-Ricobendazole, Fe-Pyrazinamide and Fe-Niclosamide, ranging from 5% to less than 23% over a wide range of potential values. The number of electrons transferred in the kinetics of ORR process is determined for all the catalysts by the following Eq. (2).

(2) $$n=\frac{4I_{disk}}{I_{disk}+\frac{I_{ring}}{N}}$$

Fig. 3c shows that most Fe-based catalysts are approaching the direct, efficient and desired 4-electron transfer mechanism, with the best performances exhibited by Fe-Ricobendazole, Fe-Pyrazinamide, Fe- Niclosamide and Fe- Guanosine.

### 3.3 Cathode electrochemical performances

**Fig. 4:**
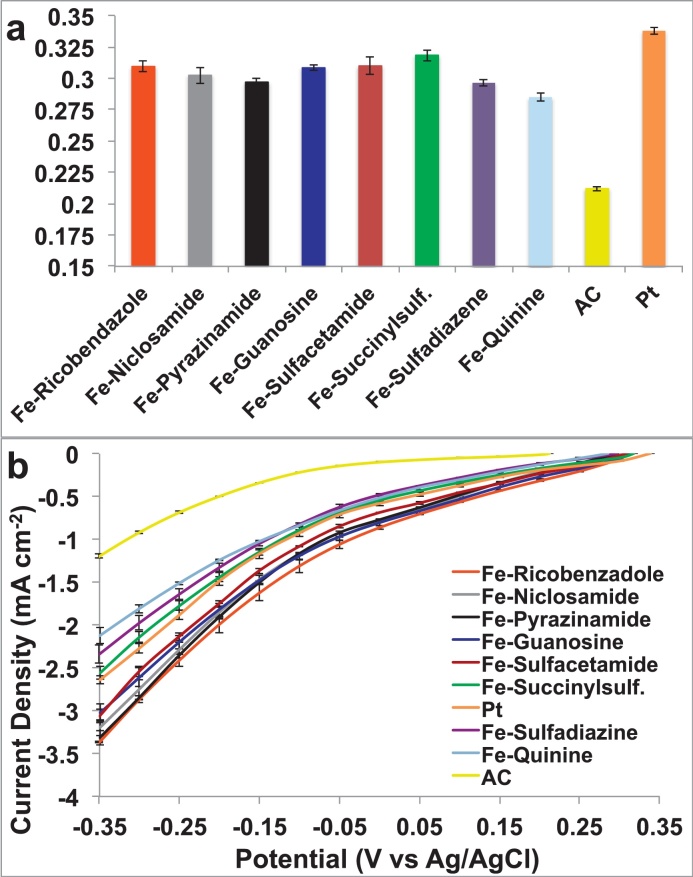
Cathode open circuit potential (OCP) (a) and linear sweep voltammetry (LSV) of cathodes in phosphate buffer (PBS) (b).

The OCP of Pt (338 ± 4 mV (vs Ag/AgCl)) was slightly higher than the Fe-N-C catalysts (303 ± 12 mV (vs Ag/AgCl)) however the level of magnitude was found to be similar and comparable along the potential investigated. Similar behavior was observed among the Fe-based catalysts. AC cathode had the lowest OCP measured in 212 ± 3 mV (vs Ag/AgCl) (Fig. 4a). This indicates that Pt and Fe-N-C catalysts had overpotentials quantified in about 230–270 mV versus the theoretical value of 570 mV vs Ag/AgCl (3 M KCl). Electrocatalytic activity was then measured for all materials of interest in a three-electrode configuration (Fig. 4b). Five of the novel synthesized catalysts Fe-Ricobendazole, Fe-Niclosamide, Fe-Guanosine, Fe-Pyrazinamide and Fe-Sulfacetamide have outperformed the electrocatalytic activity of Pt (Fig. 4b). Fe-Succinylsulfathiazole had very similar performances compared to Pt (Fig. 4b). Instead, Fe-Quinine and Fe-Sulfadiazene had much lower activity than Pt but much higher than AC (Fig. 4b). Fe-Ricobendazole, Fe-Niclosamide have been tested before with similar performances as here presented [48]. These results indicate that the presence of transition metal-based or precious-metal based catalysts significantly increases ORR activity suggesting a 4e^−^ ORR pathway. Moreover, this indicates that AC can be an excellent support for the air breathing cathode but the addition of catalyst is necessary for further increasing the performances.

### 3.4 Cathode performances in microbial fuel cell

**Fig. 5:**
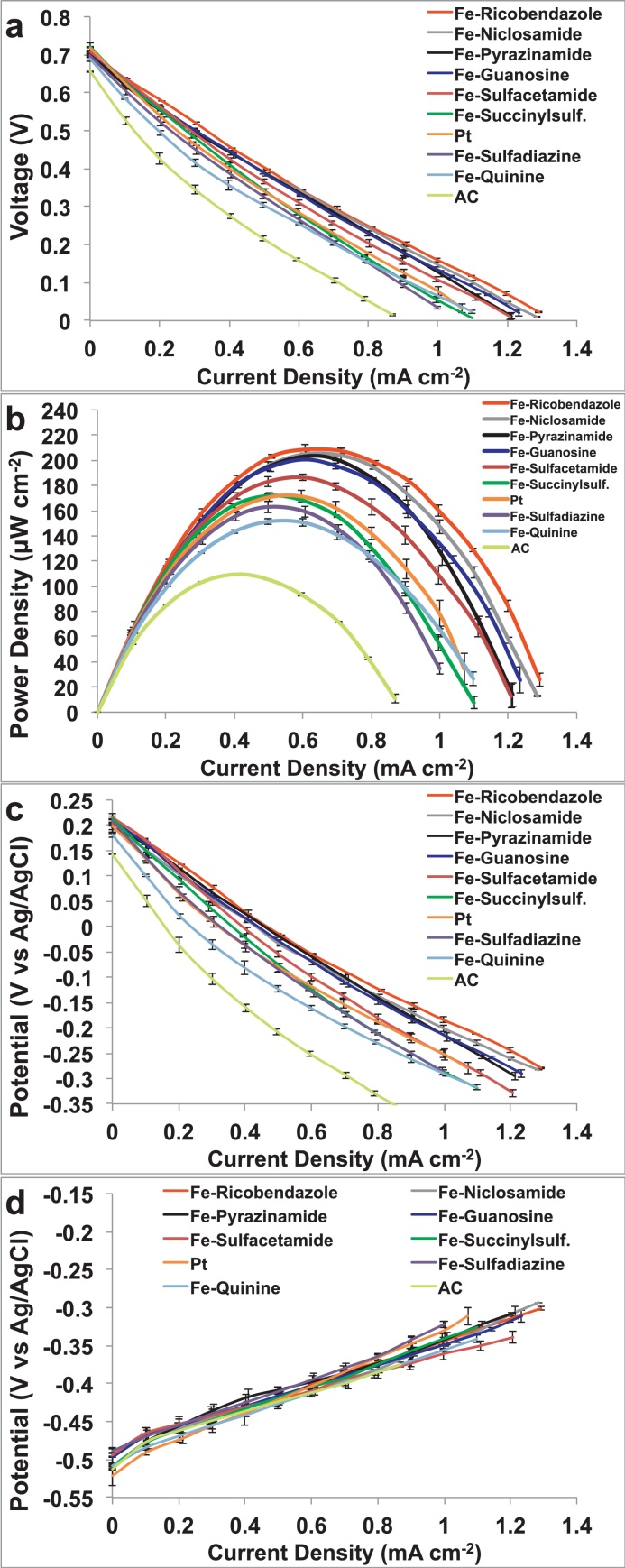
Overall MFC polarization curves (a), power curves (b) and cathode (c) and anode (d) polarization trend.

The cathodes were then incorporated into a working MFC and tested (Fig. 5). Overall polarization curves were taken after the stabilization of the OCV within 1 mV range. Platinum and iron-based cathodes showed similar OCV (715 ± 15 mV) that was roughly 60 mV superior compared to AC (656 ± 5 mV) (Fig. 5a). The MFC polarization curves (Fig. 5a) followed the trend observed during the cathode polarization curves (Fig. 4b). Fe-Ricobendazole had the highest performances followed by Fe-Niclosamide, Fe-Guanosine, Fe-Pyrazinamide and Fe-Sulfacetamide, Platinum, Fe-Succinylsulfathiazole, Fe-Sulfadiazene, Fe-Quinine and AC (Fig. 5a). Power curves showed that Fe-Ricobendazole, Fe-Niclosamide, Fe-Pyrazinamide, Fe-Guanosine and Fe-Sulfacetamide had higher performances than Pt and particularly the power densities reached: 209 ± 4 μWcm^−2^, 206 ± 3 μWcm^−2^, 202 ± 5 μWcm^−2^, 199 ± 4 μWcm^−2^, 187 ± 3 μWcm^−2^ respectively (Fig. 5b), in the case of platinum power density was found to be 171 ± 4 μWcm^−2^. Fe-Succinylsulfathiazole had power density of 172 ± 2 μWcm^−2^ comparable to Pt. Fe-Ricobendazole, Fe-Niclosamide, Fe-Pyrazinamide, Fe-Guanosine and Fe-Sulfacetamide has 22%, 20%, 18%, 15% and 9% higher power density compared to Pt. Fe-Sulfadiazine and Fe-Quinine had maximum power generation of 163 ± 4 μWcm^−2^ and 152 ± 3 μWcm^−2^ respectively that was 5% and 13% lower than Pt. Fe-Sulfadiazine and Fe-Quinine have an activity of 55% and 44% higher compared to AC (105 ± 1 μWcm^−2^) (Fig. 5b). Cathode (Fig. 5c) and anode (Fig. 5d) polarization curves obtained during the overall polarization curves showed clearly that the differences were mainly due to the different cathode utilized.

### 3.5 Similarity between catalysts tested in RRDE and catalysts integrated in air breathing cathode

**Fig. 6:**
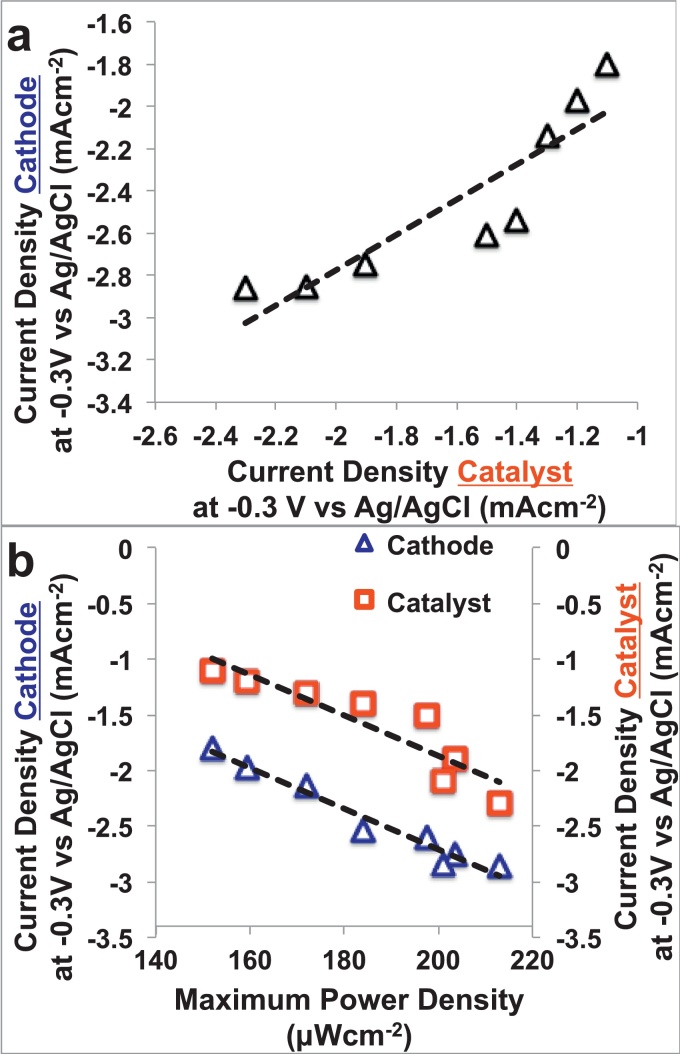
Relationship between current density measured during the RRDE tests (catalyst) and during the cathode LSV (a). Relationship between current density measured during the RRDE tests (catalyst) and during the cathode LSV with the power density achieved in MFC (b).

Interestingly, the data related on the performances (current density at −0.3 V vs Ag/AgCl) of the catalysts in the RRDE experiments correlate linearly (R^2^ = 0.81) the performances (current density at −0.3 V vs Ag/AgCl) measured in cathode polarization run in “clean” conditions in which the catalyst was incorporated within an air-breathing cathode (Fig. 6a). The current density measured in the RRDE and the one measured on the cathode during LSV run in “clean” (both measured at −0.3 V vs Ag/AgCl) conditions showed also clear relationship with the peak of power density measured in operating MFCs with R^2^ equal to 0.86 and 0.96 (Fig. 6b). Those relationships here presented for the first time in neutral media are extremely important because this suggests that the study of catalysts kinetics using RRDE disk by itself can provide an efficient and reliable way of screening catalysts and predict the best catalyst among different materials investigated without screening all the catalysts incorporated into air-breathing cathodes or in operating MFC. This methodology can be useful to certainly decrease the time necessary to identify the best performing catalysts material for MFCs.

### 3.6 Structure-to-property relationships

**Fig. 7:**
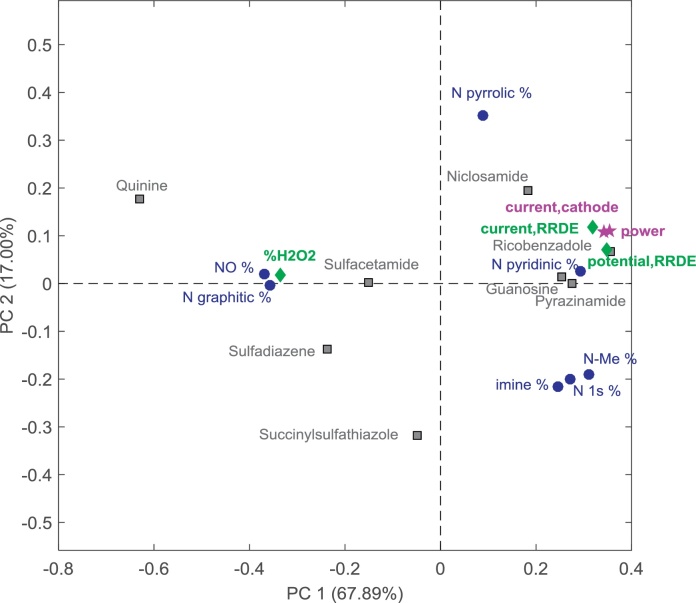
Principal Component Analysis for the Fe-N-C catalysts prepared with different organic precursors (a).

**Fig. 8:**
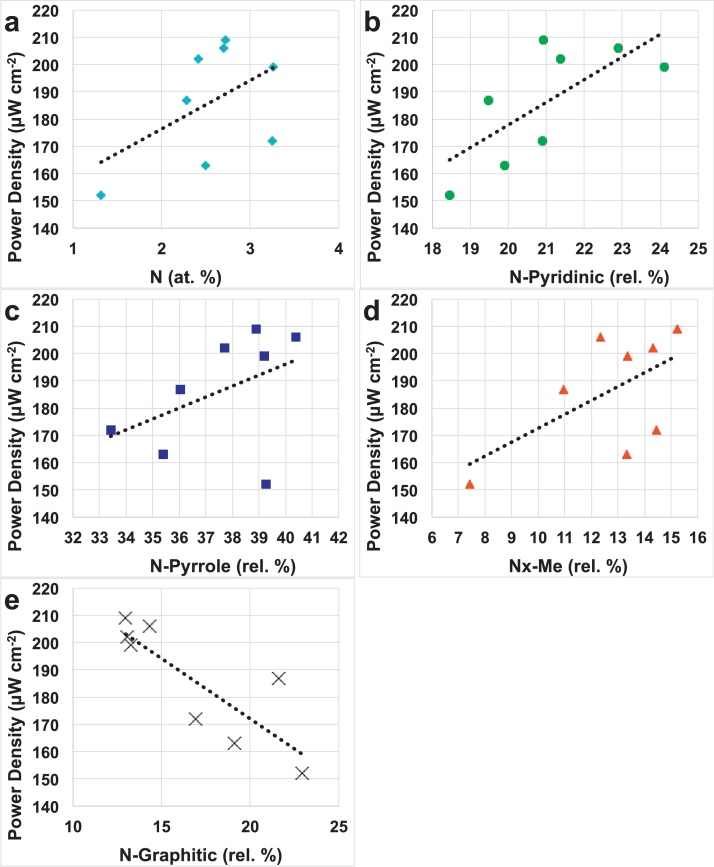
Relationship between power density and: a) total N (%); b) N-pyridinic (rel.%); c) N-pyrrole (rel.%); d) N_x_-Me (rel.%) and e) N-Graphitic (rel.%).

The principal component analysis (PCA) of the 8 different catalyst samples is presented as a biplot in Fig. 7. Data utilized in this statistical analysis were based on the power density achieved by the catalyst incorporated in the cathode of running MFCs, the current density measured during RRDE experiments at −0.3 V (vs Ag/AgCl), the H_2_O_2_ yield, the current density measured during cathode LSV experiments at −0.3 V (vs Ag/AgCl) and the surface chemistry measured through XPS. The surface chemistry considered was presented in Table 1 atomic percentage of N and relative percentage of different types of nitrogen detected. Relative distribution of individual types of nitrogen within total nitrogen should be used to study these statistical structure-to-property relationships in order to eliminate errors potentially caused by different sampling depths of N 1s, O 1s and C 1s electrons and different absolute amounts of oxygen and carbon. [56] The clustering of different variables (electrochemical vs surface chemistry) within the PCA biplot allows the discovery of interesting relationships (Fig. 7). Particularly, the power generated by the Fe-based catalysts and measures of high activity in RRDE experiments and in the cathode polarization curves have been found to be strongly correlated with the percentage of total nitrogen (N, at.%) found on the catalyst surface (Fig. 8a). Considering the different types of nitrogen detected, N-pyridinic percentage, Nx-Me%, N-pyrrolic% show strong positive correlation with power densities (Fig. 8b–d), while N-graphitic% and oxidized nitrogen groups are anti-correlated with performance (Fig. 8e). Graphitic nitrogen shows to contribute highly into hydrogen peroxide yield which is in good agreement with previous studies showing that graphitic nitrogen catalyzed the 1st step of oxygen reduction to hydrogen peroxide [57]. These relationships lead to the conclusion that the total amount of nitrogen present within the catalyst is important, but more specifically nitrogen species such as pyridinic, pyrrolic and metal coordinated nitrogen structures are contributing positively to performance of MFC in neutral media. Linear correlations of performance with pyrrolic nitrogen are in disagreement with previously reported data in which Fe-based catalyst activity in acidic or alkaline media was linearly proportional to N-pyridinic but reversely related with N-pyrrolic. [56] In fact, the presence of N-pyridinic and nitrogen coordinated to metal is generally associated with a four electrons reaction while N-pyrrolic is associated with a two electrons reaction, and the first electron mechanism is the most desired. The simultaneous benefit of N-pyridinic and N-pyrrolic for the power generation might be due to the different pH environment in which these novel catalysts are tested. There are very few in-depth studies of the non-platinum based catalyst behaviour in neutral media, so further studies will be necessary. Importantly, graphitic nitrogen which has been reported as a site which catalyzed two electrons reaction in acidic media is also proven to be not beneficial in the neutral MFC environment.

### 3.7 Cost considerations

In our previous work, it was estimated a cost of Fe-based catalyst of ≈3.4 US$ g^−1^ considering Sigma Aldrich chemicals utilized and a cost of ≈150 US$ g^−1^ for Pt/C from Alfa Aesar, Johnson Matthew Company [42]. In this current work, catalyst loading of 2 mg cm^−2^ was used for all the materials investigated. The cost analysis included only the catalyst utilized showed that a cathode surface area between 0.48 and 0.66 m^2^ was necessary to obtain 1 W of power (Table 2). Moreover, in order to obtain 1 W of power, a quantity of catalyst between 9.57 and 13.16 g should be used (Table 2). A significant difference in the cost per watt produced was identified between Fe-based materials that varied in the range of ≈32.5–44.7 US$ (Table 2). In parallel, 0.58 m^2^ were necessary to produce 1 W with a loading of 2 mg cm^−2^ and a utilization of 11.7 g of catalyst (Table 2). The total cost for W produced was ≈1754 US$ that was 39–52 times higher than Fe-based catalysts (Table 2). Due to the low power produced in microbial fuel cells systems, particular and strict attention has to be dedicated to significantly lowering the cost of materials. Once again, in this manuscript we underlined the fact that platinum cannot be successfully used as cathode catalyst for MFC application. Earth abundant metals like Fe, Co, Mn, etc. in combination with carbon-nitrogen matrix need to be used as alternative catalysts in a MFC configuration for boosting up electricity generation and dramatic reduction of the operational costs.

### 3.8 Comparison with existing literature and outlook

To the best of our knowledge, this is the fifth time overall in which catalysts kinetic using RRDE in neutral media is presented in literature [58], [59], [60], [61]. Two of the previous cases showed the RRDE data related with carbonaceous-based materials [58], [59]. Recently, RRDE data regarding iron-rich nanoparticle encapsulated nitrogen doped porous carbon [60] and Fe-phenanthroline [61] have been presented. In the first case, 6 mA cm^−2^ was achieved in neutral media at 2500 rpm in oxygen saturated conditions. A direct comparison cannot be done due to the different rpm used but those performances seem to be quite high and promising [60]. RRDE on Fe-phenanthroline showed instead at 1600 rpm a current density of 1.2 mAcm^−2^ that is roughly half compared to this current work but the solution was sparged with air and not with oxygen so a direct comparison cannot be elucidated [61]. Interestingly, once those catalysts have been used as cathode in MFCs, unexpected results have been reported. In fact, the utilization of iron-rich nanoparticle encapsulated nitrogen doped porous carbon led to a maximum power density of 123 μWcm^−2^ [60], Fe-phenanthroline [61] instead achieved 470 μWcm^−2^ while in our case the maximum power density was 209 μWcm^−2^. The main difference in those results was due to the different electrolyte, operating temperature and MFC configuration utilized.

The power measured in this work underlined that six of the in-house made catalysts have outstanding performances actually higher than Pt (even initially) that is known to be the best catalyst and the most used in microbial fuel cell [22], [23], [24]. The power density achieved in this study with the utilization of these novel catalysts is comparable to the one that we have previously reported [48]. Those values are among the highest reported for a single chamber MFC with volume greater than 0.1 L and electrolyte based on 50% 0.1 M K-PB and 50% activated sludge. In fact, the power density produced was above 200 μWcm^−2^ that is equivalent to ≈2 W m^−2^ and 4.7 W m^−3^. Despite several other platinum-free catalysts that have been used and reported in the literature [22], [23], [24], only few groups have decorated the cathode with the addition of low cost metal-based catalyst on AC support [61], [62], [63], [64], [65].

Xia et al. [62] used a mixture of Fe-EDTA and AC pressed on a stainless steel mesh achieving 158 μWcm^−2^ that was 10% higher than AC cathodes. Very recently Yang and Logan, showed Fe-phenanthroline immobilized in AC with unprecedented performances (470 μWcm^−2^) but those high values are most probably due to the electrolyte utilized with very high solution conductivity, the high operating temperature and the MFC configuration used [61]. Fe_3_O_4_ with AC and Co_3_O_4_ with AC were used Fu et al. [63] and Ge et al. [64] respectively achieving in both cases a maximum of 143 μWcm^−2^. At last, MnO_2_ catalyst mixed with AC in air cathode configuration was tested by Zhang et al. [65] with a maximum power achieved of 155 μWcm^−2^. In this work, Fe-Ricobendazole, Fe-Niclosamide and Fe-Pyrazinamide had power generated above 200 μWcm^−2^. All the catalysts presented are much cheaper than Pt and six of them outperformed Pt. Those new catalysts based on novel organic precursors have high power generation, low production cost and they seem suitable for large-scale application MFC.

## 4 Conclusions

Iron (Fe) based cathode catalysts were synthesized with eight different low cost organic precursors named Niclosamide, Ricobendazole, Guanosine, Succinylsulfathiazole, Sulfacetamide, Quinine, Sulfadiazine and Pyrazinamide. The catalysts have been investigated for oxygen reduction reaction in neutral media using RRDE and then incorporated in air-breathing cathode and then applied in microbial fuel cells. Fe-Ricobendazole, Fe-Niclosamide, Fe-Pyrazinamide, Fe-Guanosine and Fe-Sulfacetamide had higher performances than Pt. Fe-Ricobendazole, Fe-Niclosamide, Fe-Pyrazinamide had maximum power peak above 200 μWcm^−2^. Current densities recorded during RRDE experiments well correlate with the output recorded for the cathodes polarizations and the performances in operating MFC. Surface to performance relationships showed a linear dependence between power generation with N (at.%), N-pyridinic (rel.%), N-Me (rel.%), N-pyrrolic (rel.%) but it linearly decreased with N-graphitic (rel.%). Fe-based materials have a cost per watt produced that is 39–52 times cheaper than Pt.

## Figures and Tables

**Table 1:** Atomic% of N and relative% of different types of nitrogens derived from XPS N 1s spectra.

	N 1s	imine	N pyridinic	N-Me	N pyrrolic	N graphitic	NO
	%	%	%	%	%	%	%
Ricobendazole	2.7	6.8	20.9	15.2	38.9	13	5.2
Niclosamide	2.7	3.8	22.9	12.4	40.4	14.3	6.2
Pyrazinamide	2.4	8.1	21.4	14.3	37.7	13	5.5
Guanosine	3.3	5.3	24.1	13.4	39.2	13.3	4.8
Sulfacetamide	2.3	4.5	19.5	10.9	36.1	21.6	7.4
Succinylsulfath.	3.2	7.5	20.9	14.5	33.4	16.9	6.7
Sulfadiazene	2.5	4.5	19.9	13.3	35.4	19.1	7.7
Quinine	1.3	2.6	18.5	7.4	39.3	23	9.3

**Table 2:** Cost considerations for Fe and Pt based catalyst.

	μWcm^−2^	W m^−2^	m^2^ W^−1^	US$ g^−1^	g W^−1^	US$ W^−1^
Ricobendazole	209	2.09	0.48	3.4	9.57	32.5
Niclosamide	206	2.06	0.49	3.4	9.71	33.0
Pyrazinamide	202	2.02	0.50	3.4	9.90	33.7
Guanosine	199	1.99	0.50	3.4	10.05	34.2
Sulfacetamide	187	1.87	0.53	3.4	10.70	36.4
Succinylsulfat.	172	1.72	0.58	3.4	11.63	39.5
Sulfadiazene	163	1.63	0.61	3.4	12.27	41.7
Quinine	152	1.52	0.66	3.4	13.16	44.7
Platinum	171	1.71	0.58	150	11.70	1754.4
